# Supplementary material for: An integrated view on the uncertainties of sea-level rise, hazards and impacts, and adaptation
Source: Camb Prism Coast Futur. 2025 Jun 16;3:e13. doi: 10.1017/cft.2025.10003 (PMC12337580; doi:10.1017/cft.2025.10003)
Supplement: Hermans et al. supplementary material [file S2754720525100036sup001.docx]

**Supplementary Information**

**Table S1.** Representative selection of recent peer-reviewed research (published in 2020 or later) related to future sea-level rise, its hazards and impacts, and adaptation, led by authors from Dutch institutes.

| **Sea-level projections** | | |
| --- | --- | --- |
| **Authors** | **Topic** | **Institute* of first author** |
| Hermans et al. (2020) | [High-resolution SLR projections for Northwestern Europe](https://doi.org/10.1007/s00382-019-05104-5) | NIOZ/TUD |
| Van Westen et al. (2020) | [High-resolution SLR projections for the Carribean](https://doi.org/10.1038/s41598-020-71563-0) | UU |
| Lambert et al. (2020) | [Wave contribution to changes in extreme sea levels](https://doi.org/10.1088/1748-9326/ab8336) | UU |
| Muis et al. (2020) | [High-resolution dataset of extreme sea levels, including projections](https://doi.org/10.3389/fmars.2020.00263) | VU/  Deltares |
| Van Westen & Dijkstra (2021) | [Global mean SLR based on eddy-resolving models](https://www.science.org/doi/10.1126/sciadv.abf1674) | UU |
| Karabil et al. (2021) | [Landwater storage contribution to regional SLR](https://doi.org/10.3389/feart.2021.627648) | UU |
| Hermans et al. (2021) | [Global mean SLR based on CMIP6 models](https://doi.org/10.1029/2020GL092064) | NIOZ/TUD |
| Frederikse et al. (2021) | [Changes in extreme sea levels focused on high Antarctic ice sheet melt](https://doi.org/10.1038/s41467-019-14049-6) | UU |
| Lambert et al. (2021) | [Dependence between regional SLR components](http://dx.doi.org/10.1029/2020EF001825) | UU |
| Hermans et al. (2022) | [Changes in seasonal sea-level cycle Northwestern Europe](https://doi.org/10.1175/JCLI-D-21-0636.1) | NIOZ /TUD |
| Van de Wal et al. (2022) | [High-end global mean SLR projections](https://doi.org/10.1029/2022EF002751) | UU |
| Slangen et al. (2022) | [Family trees of SLR projections & timing](https://doi.org/10.1029/2021EF002576) | NIOZ |
| Slangen et al. (2023) | [Review of evolution of SLR projections](https://doi.org/10.1017/cft.2022.8) | NIOZ |
| Hermans et al. (2023) | [Timing of changes in extreme sea levels](https://doi.org/10.1038/s41558-023-01616-5) | NIOZ/UU |
| Malagón-Santos et al. (2023) | [Statistical projections of ocean dynamic sea-level change](https://doi.org/10.5194/os-19-499-2023) | NIOZ |
| Van der Linden et al. (2023) | [Antarctic basal melt contribution to SLR](https://doi.org/10.5194/tc-17-79-2023) | KNMI |
| Muis et al. (2023) | [Projections of storm surges using high-resolution climate models](https://doi.org/10.1029/2023EF003479) | VU/  Deltares |
| Jesse et al. (2024) | [Ocean dynamic sea-level change in the North Sea](https://iopscience.iop.org/article/10.1088/1748-9326/ad33d4) | KNMI/UU |
| Le Bars et al. (in review) | [Observational constraints on projections of ocean dynamic sea-level change](https://doi.org/10.5194/egusphere-2024-2872) | KNMI |
| Le Bars & Drijfhout (in review) | [Connecting sea-level projections to observed sea-level rise](https://essopenarchive.org/doi/full/10.22541/essoar.173282288.84101656/v1) | KNMI |
| **Hazards and impacts** | | |
| **Authors** | **Topic** | **Institute of first author** |
| Zhu et al. (2020) | [Saltmarsh resilience to SLR](https://doi.org/10.1002/lno.11249) | NIOZ |
| Zheng et al. (2021) | [Impact wind-waves & SLR on a sandy estuarine shoal in the Western Scheldt](https://doi.org/10.1002/esp.5207) | Deltares/  TUD |
| Bamunawala et al. (2021) | [Projections of shoreline change along inlet-interrupted coastlines](https://doi.org/10.1038/s41598-021-93221-9) | IHE/UT |
| Elmilady et al. (2021) | [Response of intertidal shoals to SLR](https://doi.org/10.1029/2021JF006152) | IHE/TUD/  Deltares |
| Nienhuis & van de Wal (2021) | [Projections of SLR impact on global delta land loss](https://doi.org/10.1029/2021GL093368) | UU |
| Van IJzendoorn et al. (2021) | [Vertical dune toe translation on prograding coasts compared to SLR](https://doi.org/10.1038/s41598-021-92150-x) | TUD |
| Huismans et al. (2022) | [Response of Wadden Sea intertidal flats to SLR](https://doi.org/10.1016/j.ocecoaman.2021.105969) | TUD/Deltares |
| Koks et al. (2022) | [Storylines of SLR impacts on infrastructure](https://doi.org/10.1080/23789689.2022.2142741) | VU |
| Lodder et al. (2022) | [Sediment transport Wadden Sea in response to SLR](https://doi.org/10.1016/j.ocecoaman.2022.106067) | TUD/  RWS |
| Vader et al. (2023) | [Assessment of remaining life of storm surge barriers under SLR scenarios](https://doi.org/10.1080/15732479.2023.2177874) | Deltares/  TU Delft |
| Nienhuis et al. (2023) | [Review of SLR impacts on river deltas](https://doi.org/10.1146/annurev-earth-031621-093732) | UU |
| Leuven et al. (2023) | [Response of peak water levels in tidal channels to SLR](https://doi.org/10.1029/2022JC019578) | WUR/Royal HaskoningDHV |
| Lodder et al. (2023) | [Results of the Coastal Genesis 2 research programme](https://doi.org/10.1016/j.ocecoaman.2023.106499) | TUD/RWS |
| Portos-Amill et al. (2023) | [Inlet expansion and barrier drowning under sea-level rise](https://doi.org/10.1029/2022JF007010) | UT |
| Aschenneller et al. (2024) | [Changing sea level & effect on shorelines Terschelling](https://doi.org/10.5194/nhess-24-4145-2024) | UT |
| Siemes et al. (2024) | [Impact of local interventions in estuaries under climate change, including SLR](https://doi.org/10.1029/2023JF007595) | UT |
| Zamrsky et al. (2024) | [Global impact of SLR on coastal fresh groundwater](https://agupubs.onlinelibrary.wiley.com/doi/10.1029/2023EF003581) | UU |
| Van de Wal et al. (2024) | [Review of SLR impacts & consequences in Europe](https://doi.org/10.5194/sp-3-slre1-5-2024) | UU |
| **Adaptation** | | |
| **Authors** | **Topic** | **Institute of first author** |
| Tiggeloven et al. (2020) | [Global benefit–cost analysis of coastal flood adaptation](https://doi.org/10.5194/nhess-20-1025-2020) | VU |
| Haasnoot et al. (2020) | [Adaptation strategy NL to SLR in relation to high Antarctic ice melt](https://doi.org/10.1088/1748-9326/ab666c) | UU/  Deltares |
| Van Alphen et al. (2020) | [Adaptation strategies & lessons from long-term planning in NL & Bangladesh](https://doi.org/10.1080/02508060.2021.1911069) | Staff DC |
| Haasnoot et al. (2021) | [Long-term SLR & necessity of commitment to adaptation](https://doi.org/10.1016/j.crm.2021.100355) | UU/  Deltares |
| Haasnoot et al. (2021) | [Pathways to coastal retreat in response to SLR](https://doi.org/10.1126/science.abi6594) | UU/  Deltares |
| Duijndam et al. (2021) | [Relation between SLR-related hazards & migration](https://doi.org/10.1002/wcc.747) | VU |
| Timmerman et al. (2021) | [Ecological consequences of SLR and adaptation strategies in the Wadden](https://doi.org/10.1016/j.ocecoaman.2021.105674) | UU |
| McEvoy et al. (2021) | [Assessment of how European countries are planning to adapt to SLR](https://doi.org/10.1016/j.ocecoaman.2020.105512) | Deltares |
| Tiggeloven et al. (2022) | [Coastal flooding adaptation & role of foreshore vegetation](https://doi.org/10.1111/jfr3.12790) | VU |
| Cox et al. (2022) | [Effectiveness of sedimentation-enhancing strategies for river deltas and estuaries](https://doi.org/10.1016/j.gloplacha.2022.103796) | UU |
| Dunn & Minderhoud (2022) | [Effectiveness sedimentation strategies in adaptation to relative SLR of Mekong delta](https://doi.org/10.1038/s43247-021-00331-3) | UU |
| De Bruijn et al. (2022) | [Rhine-Meuse delta protection strategies against SLR & river discharge](https://doi.org/10.1111/jfr3.12782) | Deltares |
| Van Alphen et al. (2022) | [Potential consequences of accelerated SLR & adaptive strategies in The Netherlands](https://doi.org/10.3390/w14101527) | Staff DC |
| Van den Hurk et al. (2022) | [Science-policy challenges of SLR adaptation in Northwestern Europe](https://doi.org/10.1016/j.crm.2022.100403) | Deltares/  VU |
| Van Valkengoed et al. (2022) | [Relationships between climate change perceptions and climate adaptation](https://link.springer.com/article/10.1007/s10584-022-03338-7) | RUG |
| Van Valkengoed et al. (2024) | [Risk perception & efficacy beliefs in climate adaptation](https://doi.org/10.1111/risa.14193) | RUG |
| Van Ginkel et al. (2022) | [Assessment of socio-economic tipping points under different SLR scenarios and adaptation strategies](https://doi.org/10.1016/j.crm.2022.100445) | VU/ Deltares |
| Tierolf et al. (2023) | [Adaptation and migration decisions under future coastal flood risk in France](https://doi.org/10.1038/s41598-023-31351-y) | VU |
| Mortensen et al. (2024) | [Global coastal flood risk reduction using DRR](https://doi.org/10.5194/nhess-24-1381-2024) | VU |
| Aerts et al. (2024) | [Review of limits & gaps flood adaptation](https://doi.org/10.1038/s44221-024-00274-x) | VU/Deltares |
| Tierolf et al. (2024) | [Coastal adaptation and migration under SLR](https://doi.org/10.1016/j.scitotenv.2024.170239) | VU |
| Geukes et al. (2024) | [Sand nourishment for multifunctional coastal climate adaptation](https://doi.org/10.1016/j.nbsj.2024.100191) | LU |
| Haasnoot et al. (2024) | [Lessons from adaptation pathways studies](https://doi.org/10.1016/j.gloenvcha.2024.102907) | UU/Deltares |
| Van den Hurk et al. (2024) | [Summary for policymakers of European report on SLR and coastal impacts](https://doi.org/10.5194/sp-3-slre1-1-2024) | Deltares/  VU |
| Vinke-de Kruijf et al. (2024) | [Diversification of flood risk management in the Netherlands](https://doi.org/10.1111/jfr3.1302) | UT |
| Vinke-de Kruijf et al. (2024) | [Climate resilient water infrastructure and necessity of interdisciplinarity and participatory approaches](https://doi.org/10.1002/jci3.12017) | UT |
| Ter Harmsen van der Beek et al. (in review) | [Transformative decisions & adaptation pathways](https://papers.ssrn.com/sol3/papers.cfm?abstract_id=5131262) | Deltares |

*IHE = Delft Institute for Water Education; KNMI = Royal Netherlands Meteorological Institute; LU = Leiden University; NIOZ = Royal Dutch Institute for Sea Research; RUG = University of Groningen; RWS = Department of Waterways and Public Works; Staff DC = Staff of the Delta Commissioner; TUD = Delft University of Technology; UT = University of Twente; UU = Utrecht University; VU = Free University of Amsterdam; WUR = Wageningen University & Research.

**Table S2: List of workshop participants, their institutes*, and their field of expertise.**

|  | **Participant** | **Institute(s)** | **Expertise** |
| --- | --- | --- | --- |
| **1** | T.H.J. Hermans | UU | Sea level, extremes |
| **2** | D. Le Bars | KNMI | Sea level, oceanography |
| **3** | R. Rietbroek | UT | Sea level, observations |
| **4** | E. van der Linden | KNMI | Sea level, polar climate |
| **5** | R. Gelderloos | TUD | Sea level, oceanography |
| **6** | R.S.W. van de Wal | UU | Sea level, ice sheets |
| **7** | A. Slangen | NIOZ, UU | Sea level, climate |
| **8** | J.E.A. Storms | TUD | Coastal morphodynamics |
| **9** | Y. Huismans | Deltares, TUD | Salinization, intertidal basins |
| **10** | F. Diermanse | Deltares | Flood risk |
| **11** | S.G. Pearson | TUD | Coastal morphodynamics, engineering |
| **12** | K.M. Wijnberg | UT | Coastal systems, sediments |
| **13** | G. Winter | Deltares | Coastal hazards, adaptation |
| **14** | F.E. Dunn | UU | Delta processes, adaptation |
| **15** | A. Budding | WUR | Water management |
| **16** | M. Haasnoot | Deltares, UU | Climate adaptation, decision-making |
| **17** | T. Haer | VU | Equitable adaptation, climate justice |
| **18** | L.M. Kreemers | HvA | Social psychology, behavioral change |
| **19** | R.C. de Winter | Deltares | Climate impacts, adaptation |
| **20** | H. Erenstein | Min. I&W | Policy & research advisor |
| **21** | A. Roeling | Min. I&W | Policy coordinator |
| **22** | L. de Vries | Staff DC | Delta programme-manager |

*UU = Utrecht University; KNMI = Royal Netherlands Meteorological Institute; UT = University of Twente; TUD = Delft University of Technology; NIOZ = Royal Dutch Institute for Sea Research; WUR = Wageningen University & Research; VU = Free University of Amsterdam; HvA = Amsterdam University of Applied Sciences; Min. I&W = Ministry of Infrastructure and Water Management; Staff DC = Staff of the Delta Commissioner


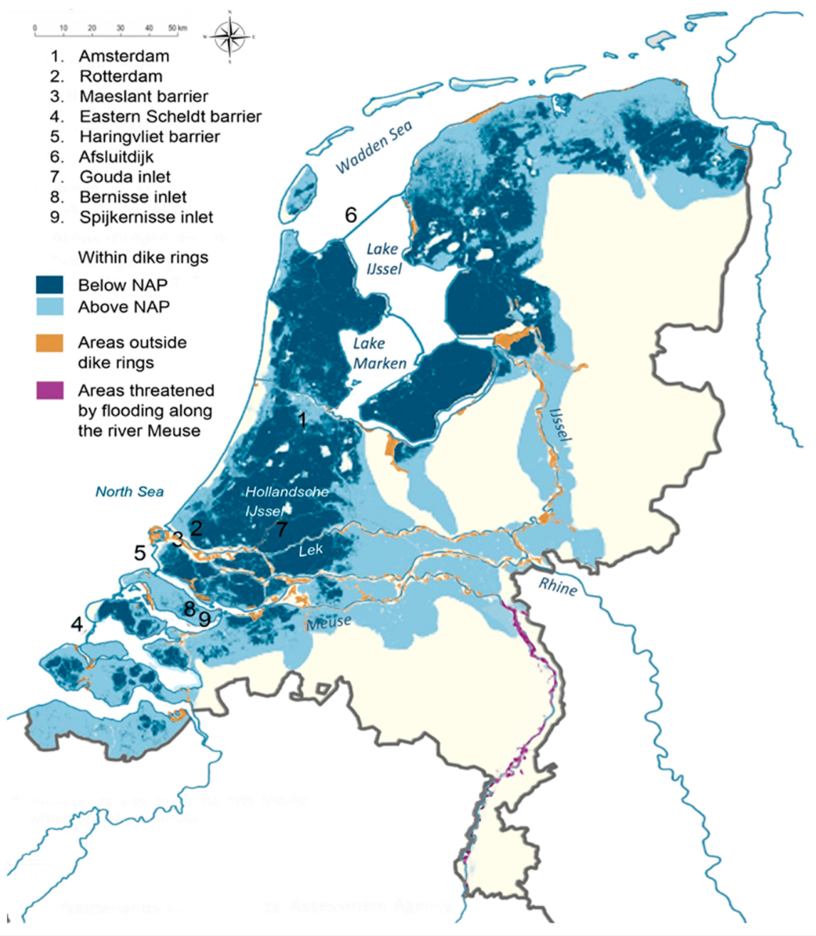


**Figure S1:** Map of the Netherlands, major seas, lakes and rivers, low-lying regions (below or above the reference plane for sea level height in the Netherlands (NAP)), and several features of the Dutch water management system. Reprinted from Haasnoot et al. (2020b).
